# Procalcitonin (PCT) and C-reactive Protein (CRP) as severe systemic infection markers in febrile neutropenic adults

**DOI:** 10.1186/1471-2334-7-137

**Published:** 2007-11-22

**Authors:** Karin SR Massaro, Silvia F Costa, Claudio Leone, Dalton AF Chamone

**Affiliations:** 1 Disciplina de Hematologia e Hemoterapia – Departamento de Clínica Médica at the Faculty of Medicine of the University of São Paulo, Brazil; 2 Pesquisa e Desenvolvimento – Departamento de Doenças Infecciosas e Parasitárias at the Faculty of Medicine of the University of São Paulo, LIM54, Brazil; 3 NUCAMP – Núcleo de Consultoria e Apoio em Metodologia de Pesquisa e Estatística, Departamento de Pediatria at the Faculty of Medicine of the University of São Paulo, Brazil; 4 Av. General Ataliba Leonel, 93 cj112, São Paulo – SP, 02033-000, Brazil

## Abstract

**Background:**

Procalcitonin (PCT) is an inflammatory marker that has been used as indicator of severe bacterial infection. We evaluated the concentrations of PCT as a marker for systemic infection compared to C-reactive protein (CRP) in patients neutropenic febrile.

**Methods:**

52 adult patients were enrolled in the study. Blood sample was collected in order to determine the serum concentrations of PCT, CRP and other hematological parameters at the onset of fever. The patients were divided into 2 groups, one with severe infection (n = 26) and the other in which the patients did not present such an infection (n = 26). Then PCT and CRP concentrations at the fever onset were compared between groups using non parametric statistical tests, ROC curve, sensitivity, specificity, likelihood ratio, and Spearman's correlation coefficient.

**Results:**

The mean of PCT was significantly higher in the group with severe infection (6.7 ng/mL versus 0.6 ng/mL – p = 0.0075) comparing with CRP. Serum concentrations of 0.245 ng/mL of PCT displayed 100% de sensitivity and 69.2% specificity. PCT concentrations of 2,145 ng/mL presented a likelihood ratio of 13, which was not observed for any concentration of CRP.

**Conclusion:**

PCT seems to be an useful marker for the diagnosis of systemic infection in febrile neutropenic patients, probably better than CRP.

## Background

Patients suffering from malignant hematological diseases present long periods of profound neutropenia which are a result of chemotherapy and of other factors associated to the disease. During this neutropenic state, the risk of infection is high [1]. The morbidity and mortality associated to infection acquired after aggressive chemotherapy remain the greatest clinical problem suffered by these patients. Considering that fever in neutropenic patients is an emergency condition, that there is initially a paucity of clinical information and that obtaining results for microbiological tests may take considerable time, a new clinical laboratory test that would assist the physician in decision making as to the therapeutic conduct to be adopted in such cases would be invaluable.

Two such laboratory parameters that fit this purpose are C-reactive protein (CRP) and interleukin-6 (IL-6) [2]. CRP is an acute phase protein used mostly for as a biochemical inflammatory marker in cancer, however, its concentration does not significantly increase until 24 to 48 hours after the onset of inflammation. Serum concentrations of CRP are proportional to the degree of tissue damage and the activity of the basal malignant disease [3-6]. IL-6 concentration presents a low specificity and furthermore, its determination is costly [6].

Procalcitonin (PCT) is a peptide composed of 116 aminoacids, also known as the prohormone of calcitonin, and is well documented that its concentration increases in patients with bacterial meningitis or sepsis, however, its main lieu of synthesis and its function have not been fully elucidated yet [7-12].

The association of PCT high concentration with infection may be useful for diagnosis patients with febrile neutropenia, especially in those with systemic infections, and this supports the need of evaluating PCT as a marker for severe systemic infection and to compare it to CRP, which is currently the most used marker for this purpose.

## Methods

In order to determine whether PCT can be used as a marker for bacterial infection in febrile neutropenic patients, a cohort study was drawn up, composed of a sequential convenience sample of 52 adult inpatients with neutropenia hospitalized at the Hematology Infirmary at the Hospital das Clínicas of the Faculty of Medicine at the University of São Paulo, from August 2004 to September 2006. All patients enrolled in the study suffered from a malignant hemopathy, were severely neutropenic, either due to chemotherapy, or as a consequence of the disease and all patients were febrile.

Neutropenia was defined as a neutrophil count of less than 500/mm^3 ^or less than 1,000/mm^3 ^with an expected decline to 500/mm^3 ^[1]. Fever was defined as a single body temperature peak over 38.3°C or a sustained body temperature of more than 38.0°C for over 1 hour, not being a consequence of the administration of blood-derived products [1].

Patients whose febrile states were clearly associated to the infusion of blood-derived products were excluded from the study as were those who had received antibiotic therapy during a 12 hour period immediately before the collection of a blood sample for use in the study.

With the diagnosis of a febrile state, 3 to 5 mL of blood were collected from patients participating in the study by forearm venipuncture or from a central venous catheter. The blood was collected in dry tubes and was then centrifuged for 15 minutes at 3400 rpm up to 60 minutes after collection. The serum obtained from each sample was stored at -20°C until the moment of analysis.

All patients were evaluated as to the existence of a site of infection using clinical and microbiological data when they developed fever and received antimicrobial therapy according to the standard procedure used for patients with febrile neutropenia [1].

The criteria used for the diagnosis of infection and colonizers were those according to the CDC [13]. The diagnosis of Coagulase negative Staphylococcus (CoNS) bacteremia was considered when a patient presented multiple isolates presenting the same antimicrobial susceptibility profile from at least 2 different blood cultures collected at different time periods. Sepsis was defined as SIRS (systemic inflammatory response syndrome) with clinical signs of any underlying infection with or without growing microorganisms from any culture of any biological fluid [13]. Severe sepsis was considered when fever was accompanied by signs of tissue hyperperfusion such as oliguria, metabolic acidosis or deterioration of the level of consciousness [14]. Fever of Undetermined Origin (FUO) was considered when no cause of fever could be established after three days of hyperthermia [15].

A complete clinical follow-up was carried out for each patient from the onset of neutropenia until clinical resolution (death or discharge), by means of a standardized clinical and laboratory data file. This system had the objective of classifying the patients into groups according to diagnosis of infection. The clinical data recorded were physical signs, hematology and chemistry parameters, results of blood, urine and tissue secretion cultures, radiographs and CT scans of the thorax, paranasal sinuses and abdomen, when necessary.

The patients were classified clinical and microbiologic criteria into one of these two groups:

Group 1 – Patients with severe infection (fever + positive blood culture for bacteria or fungi) or clinical signs of sepsis as well as patients with a proven fungal infection, n = 26.

Group 2 – Patients with FUO, with or without a known colonizer, or localized bacterial infection in a single site (for instance: abscess, pneumonia, skin), as proven by a complementary test.

### Data interpretation

The correlation between clinical condition and PCT concentration of was considered according to the following criteria proposed by Giamarellou *et al*. (2004) [12]:

[PCT] < 0.5 ng/mL – Fever of Undetermined Origin (FUO)

0.5 ng/mL ≤ [PCT] < 1.0 ng/mL – Localized infection

1.0 ng/mL ≤ [PCT] < 5.0 ng/mL – Bacteremia or probable sepsis

[PCT] ≥ 5.0 ng/mL – Severe sepsis

Blood samples for PCT and CRP analysis were collected either before or up to 12 hours after the initiation of antimicrobial therapy.

### PCT determination

Serum concentrations of PCT were determined in duplicate by an immunoluminometric assay (Brahms, PCT-LIA, Berlin, Germany) that demands only 20 μL of serum as sample. The lowest concentration of PCT detectable by this technique is 0.08 ng/mL.

### CRP determination

The quantitative determination of CRP was carried out using a kit based on nephelometry (Dade-Behring N High Sensitivity CRP). The reference range for normal individuals supplied by the kit manufacturer is ≤3, 5 mg/L.

### Statistical analysis

The levels of PCT and CRP were compared between the 2 groups. Statistical analysis was carried out using Student's t-test for parametric or Mann-Whitney test for medians of non parametric data and Fischer's Exact Test for the comparison of proportions. The Spearman's correlation coefficient between PCT and CRP was calculated for each group. The sensitivity, specificity, positive and negative predictive values, the likelihood ratio and the area under the ROC (Receiver Operator Characteristic) curve were calculated for both PCT and CRP.

Finally, a Wald Backward Stepwise Logistical Regression Analysis for multiple variables was carried out using as independent variables the concentrations of PCT, PCR, granulocyte and platelet count, and using as a dependent variable in the first analysis the presence of systemic infection and in the second analysis the outcome of death. The values were calculated with the respective 95% confidence intervals and a level of significance of α = 5%. For statistical analysis the data was stored in an MS-Excel^® ^2003 spreadsheet and later processed using the software Instat^® ^and Prism4^® ^Graphpad and SPSS^® ^version 12.0[16,17].

## Results

The distribution of patients as to sex, age, recentness of diagnosis versus re-incidence or non-responsiveness of disease, granulocyte and platelet count, and hemoglobin concentration were homogeneous throughout both groups. The mean age was of 40.8 ± 16.0 years in the group with severe infection (group 1) and 40.0 ± 18.4 in the group without severe infection (group 2). The sex ratio in each group was 65.4% and 73.1% of males in group 1 and group 2, respectively. As to the presence of recent diagnosis we observed 73.1% and 65.4% for both groups, respectively. For groups 1 and 2 the mean granulocyte counts were 77 ± 137/mm^3 ^and 86 ± 140/mm^3^, respectively, the mean platelet counts were 37,961 ± 28,593/mm^3 ^and 35,962 ± 21,510/mm^3^, and the mean hemoglobin concentrations were 8.0 ± 2.4 g/dL and 8.5 ± 1.6 g/dL, respectively. The frequency of deaths associated to the febrile episodes for group 1 were of 11/26 and 2/26 for group 2 (p = 0.009). All patients were suffering from malignant hemopathies. Amongst the diagnoses were acute leucoses (40/52), chronic myelomonocytic leukemia (2/52), Hodgkin's Lymphoma (1/52) and non-Hodgkin Lymphoma (9/52). The infectious diagnoses of each patient can be seen in Table 1. Seventy three blood cultures were positive among the neutropenic febrile patients, Gram-negative was the most frequent agent isolated responsible for 38 (54%) of positive cultures followed by Gram-positive 34(48%) and fungi 1 (1.4%). Among the Gram-negative the most frequent agents identified were *Enterobacter aerogenes *(36%) and *Klebsiella pneumoniae *(30%) and among Gram-positive, coagulase negative Staphylococcus was responsible for 28 (82%) of positive blood culture. Two lung cultures and 01 urine culture was positive respectively for *S. aureus *and *Cryptococcus neoformans *and *Acinetobacter baumannii*. The concentrations of PCT and CRP in neutropenic febrile adults with severe infection (group 1) and without severe infection (group 2) can be seen in Table 2.

**Table 1:** Infectious diagnosis of the 52 febrile neutropenic patients.

**Diagnosis**	**Number of patients (%)**
FUO* with known contaminant	4(7,7)
**FUO without known contaminant**	14(26,9)
UTI**	1(1,9)
**Cellulitis (soft tissue infection)**	1(1,9)
**Catheter infection**	1(1,9)
**Pseudomembranous colitis and soft tissue infection**	1(1,9)
**Neutropenic colitis**	1(1,9)
**Pneumonia**	2(3,8)
**Dental abscess**	1(1,9)
**Pulmonary cryptococcosis**	1(1,9)
**Bloodstream infection**	5(9,6)
**Sepsis or severe sepsis**	12(23,1)
**Septic shock**	2(3,8)
Septic shock and MOF***	6(11,6)
**Total**	52(100,0)

*FUO: Fever of Undetermined Origin; **UTI: Urinary Tract Infection ; ***MOF: Multiple Organ Failure.

**Table 2:** Procalcitonin (PCT) and C-reactive protein (CRP) concentrations in adult febrile neutropenic patients according to presence or absence of systemic infection.

**Markers**	**Group with severe systemic infection (n = 26)**	**Group without systemic infection (n = 26)**	**Ststistical Significance**
**PCT(ng/mL) median (min-max)**	2,30 (0,27–50,96)	0,49 (0,08–2,15)	P = 0,0003(Sig)*
**CRP(mg/L) median (min-max)**	101,5 (5,2–341,0)	87,8 (19,5–546,0)	P = 0,9198(NS)*

* Mann-Whitney test

As can be seen in tables 3 and 4, serum levels of 0.245 ng/mL (≈0.5) of PCT are able to identify 100% of the febrile neutropenic patients with systemic infection and enabled to discard of almost one third of the patients without severe systemic infection.

**Table 3:** Verosimilarity ratio (VR) of different cut-off points of procalcitonin (PCT) concentration for the diagnosis of systemic infection in patients with febrile neutropenia.

** [PCT] ng/mL**	**With systemic infection (sensitivity)**	**Without systemic infection (1 - specificity)**	Likelihood ratio*	**(95% CI)**
**0,245**	26 100%	18 69,2%	1,44	(Incalculable*)
**0,275**	24 92,3%	18 69,2%	1,33	(1,01 to 1,76)
**0,490**	22 84,6%	13 50,0%	1,69	(1,11 to 1,76)
**1,160**	15 57,7%	4 15,4%	3,75	(1,44 to 9,79)
**1,730**	14 53,8%	2 7,7%	7,00	(1,76 to 27,78)
**2,145**	13 50,0%	1 3,8%	13,00	(1,83 to 92,29)
**2,390**	13 50,0%	0 0%	(Incalculable*)	
**TOTAL**	26 100,0%	26 100,0%		

* Impossible to calculate, for the values of sensitivity or of 1 - specificity correspond to 100% of the sample with or without the disease.

**Table 4:** Verosimilarity ratio (VR) of different cut-off points of C-reactive protein (CRP) concentration for the diagnosis of systemic infection in patients with febrile neutropenia.

**[CRP] mg/L**	**Group with systemic infection (n = 26)**	**Group without systemic infection (n = 26)**	**Likelihood ratio**	**(95% CI)**
**21,30**	23 88,5%	25 96,2%	0,92	(0,79 to 1,08)
**40,00**	18 69,2%	24 92,3%	0,75	(0,56 to 0,99)
**72,00**	16 61,5%	15 53,8%	1,07	(0,68 to 1,67)
**140,00**	11 42,3%	7 26,94%	1,57	(0,72 to 3,41)
**173,00**	5 19,2%	4 15,4%	1,25	(0,38 to 4,14)
**214,50**	1 3,8%	1 3,8%	1,00	(0,06 to 15,15)
**TOTAL**	26 100,0%	26 100,0%		

In these tables, the likelihood ratio is also different between PCT and CRP, which suggests that among patients who present PCT concentrations of 2.145 ng/mL, the chance of a systemic infection occurring is of 13 to 1, whilst no probability of this magnitude was observed for different cut-off levels or CRP.

**Figure 1:**
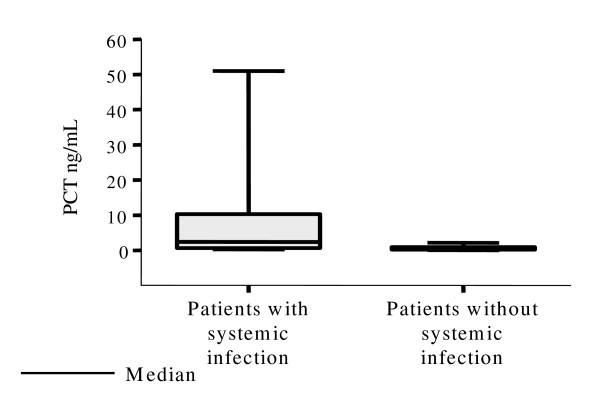
Distribution of the serum concentrations of procalcitonin (PCT) in neutropenic febrile patients according to presence or absence of systemic infection.

**Figure 2:**
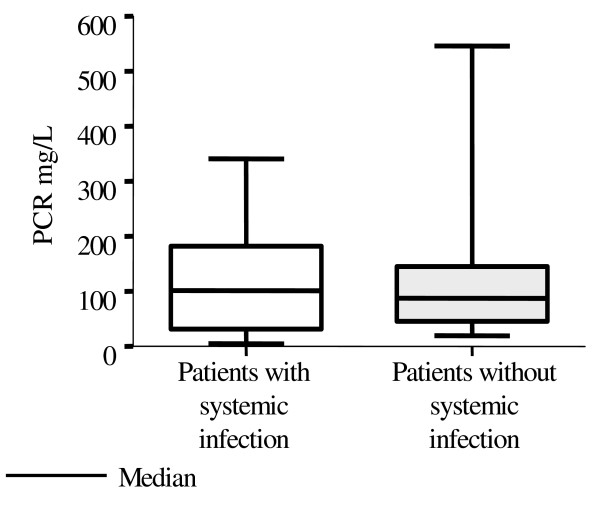
Distribution of serum concentrations of C-reactive protein (CRP) in febrile neutropenic patients according to presence or absence of systemic infection.

**Figure 3:**
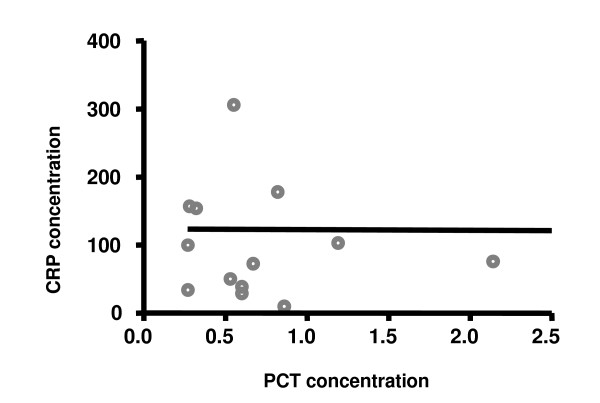
Correlation between the serum concentrations of C-reactive protein (CRP) and procalcitonin (PCT) in febrile neutropenic patients and systemic infection. Spearman correlation coefficient r_s _= 0.066 (p = 0.746, not significant)

Figures 1 and 2 show that the medians and dispersion of PCT and CRP concentrations were statistically differents in patients with and without systemic infection. There was no correlation between serum levels of PCT and CRP, as can be seen in figure 3, in both groups with and without systemic infection. This was confirmed by the Spearman Correlation Coefficient that failed to indicate statistical significance in both groups.

**Figure 4:**
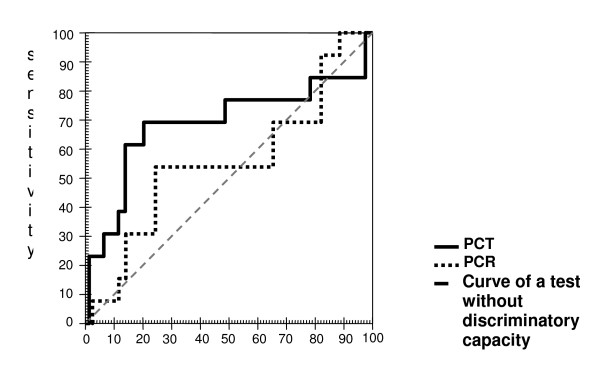
Comparison between the procalcitonin (PCT) and C-reactive protein (CRP) ROC curves in febrile neutropenic patients, according to death* related to the febrile event as an outcome. *Deaths: 13. *Not Deaths: 39. 1 - specificity. **significant. PCT – area under the curve: 70.8% (95% CI: 50.9 to 90.7)**. PCR – area under the curve: 57.8% (95% CI: 38.4 to 77.2)

The ROC curves for PCT for the diagnosis of infection and prognosis can be seen in figure 4. The area under the curve is substantially different between PCT and CRP.

Calculated *a posteriori*, considering the sample size of 52 patients (two groups of 26), the observed areas under the ROC curve of 79.1% for PCT and of 50.0% for CRP and the Spearman (rank) coefficients between PCT and CRP of 0.07 for Group 1 and of 0.08 Group 2, the power of the test (1 - β) was of 0.80 to discriminate the areas difference.

The only independent variable that remained associated to severe infection in the Wald Backward Stepwise Multivariate Logistic Regression model was PCT (OR = 2.49; 95% CI = 1.18–5.27; p = 0.016).

In the prognosis model of Multivariate Logistic Regression, the only significant association observed with death was the platelet count (OR = 1.0; 95% CI = 1.0–1.0; p = 0.031).

## Discussion

Febrile neutropenia is a medical emergency that calls for a precocious diagnosis and the administration of antibiotics as soon as possible. The patient's clinical, microbiological and radiological data usually fail in revealing the origin of the fever. Even though it is known that PCT is also produced to some extent by leukocytes [18], studies have shown that PCT may be useful as a marker in neutropenic patients [7,8,12,19,20]. Al Nawas *et al*. (1996) have shown that PCT is increased in 25 patients with neutropenia and sepsis [8]. Giamarellos-Bourboulis et al., in a prospective study with 115 febrile neutropenic adults, revealed that PCT might be a useful diagnostic tool for the early detection of a systemic infection in this population [7].

In our study the following variables: sex, age, recentness of diagnosis versus re-incidence or non-responsiveness of disease, granulocyte and platelet count and hemoglobin concentration were similar between groups (p > 0.05). The rates of death related to the febrile event was shown to be strikingly different between both groups, with 11 deaths in group 1 (systemic infection) in contrast to only 2 deaths in group 2 (p = 0.009). As to the basal hemopathies, over 2/3 of all patients were suffering from some form of leukemia.

The most common causes of fever in our patients were FUO, followed by sepsis. The most frequently isolated microorganisms were *Staphylococcus sp. *followed by *Enterobacter sp. *and *Klebsiella sp*.

In spite of the sample size (n = 52) and of the single determination of PCT at the onset of fever, the increased values of PCT were able to indicate infection already on the first day of fever in group 1. These neutropenic patients with severe infection (diagnosed clinically and/or microbiologically) presented high concentrations of PCT, but CRP concentrations evinced no correlation with severe infection.

The mean PCT concentrations were statistically different between both groups already at the beginning of the febrile state, with PCT being much more elevated among patients with severe infection. This was not observed in the serum concentrations of CRP. For patients with neutropenia who did not have systemic infection (group2), that is, those who were diagnosed with FUO or localized infection presented low concentrations of PCT (0,08–2,15) that were highly significant (p = 0,0003) and CRP concentrations that did not greatly differ statistically from those found in group 1 (19,5–546,0) (p = 0,9198).

It can be seen in these cases that there is no correlation between PCT and CRP, that is, the variations in PCT concentration are not concomitant with the variations in CRP and the results further indicate that the PCT concentrations change more precociously than those of CRP in response to an infection. Also, there is no correlation between PCT versus PCR values in the group without systemic infection. Because PCT concentration changes more rapidly than CRP in response to infection (bacterial and/or fungal), a diagnostic decision can be taken more precociously, which would aid in speeding up choosing the therapeutic conduct.

It can be seen, from the ROC curve, that CRP cannot be used as a diagnostic or prognostic marker in febrile neutropenia. The area under the ROC curve for PCT is of 79% (CI between 67% and 91%), in contrast to 50% for CRP (CI between 34% and 67%) for diagnosing systemic infection in the groups studied.

Giamerellou et al. clearly showed in an important multicentre study concerning febrile neutropenia that PCT levels were elevated significantly in patients with severe sepsis compared to bacteremia (n = 158) [12]. A PCT cut-off value of 1.0 ng/mL could indicate bacteraemia, while severe sepsis could be shown with a level over 5.0 ng/mL. In this present study, the cut-off points used for PCT were 0.25 ng/mL; 0.5 ng/mL, 1.0 ng/mL and 2.0 ng/mL. Using values of 0.25 ng/mL, it was possible to discriminate between presence or absence of systemic infection with a sensitivity of 100% and a specificity of 30.8%. When the cut-off point used was of 0,49 ng/mL, the sensitivity and specificity obtained were of 85% and 50%, respectively. There were no satisfactory cut-off values for CRP.

The PCT ROC curves for evolution to death as a result of systemic infection limit an area under the curve statistically different than that expected by chance, whereas this is not the case for CRP. Furthermore, specifically for infection, one can see by means of the confidence intervals that there is a statistically significant difference between the PCT ROC curve and the CRP ROC curve. The main problem as to the determination of PCT is the ample variation of the results obtained that do not follow a Gaussian distribution, which thus makes it more difficult to determine a cut-off point with an optimum sensitivity and specificity adequate enough to indicate an infection. These data corroborate those obtained by Giamarellos-Bourboulis *et al*. (2001), who also found this distribution [7]. Even though a PCT concentration that fulfills adequate sensitivity and specificity and considering that a sensitivity of 73% has been proposed by other authors as satisfactory for PCT [7,21], we could assume that concentrations of 0.49 ng/mL, or better 0.5 ng/ml, with 85% sensitivity and 50% specificity might be useful for the differential diagnosis between a disseminated infection (bacteremia, sepsis and its intermediate stages) and a non disseminated infection (FUO and localized infection).

Cut-off values of more than 2,0 ng/mL, which are suggested by the manufacturer for immunocompetent patients would increase the specificity to 96.2%, however, the sensitivity would drop to 53%, which would imply that a severe infection might not be considered in 1 of each 2 really infected patients. As the PCT concentrations rise, sensitivity decreases and specificity increases, to the point which leads us to assume that values >2.0 ng/mL in a single patient might indicate severe sepsis.

Using logistical regression, only PCT remained in the model as associated to the presence of severe infection. Thus, CRP concentration was not able to distinguish between presence or absence of a disseminated infection, perhaps because of the contribution of the basal malignant disease to the production of CRP [22]. Recently, Schuttrumpf et al. evaluated PCT plasma concentrations prospectively in 111 patients with a hemato-oncological condition with a CRP concentration >8 mg/L. Median CRP concentrations did not differ significantly between groups of patients with and without infection. However, PCT concentrations were higher in patients with infection than in those without infection and contributed significantly to the differential diagnosis of elevated CRP concentrations in patients with hemato-oncological conditions [23].

The prognostic significance of PCT has already been demonstrated in studies with immunocompetent adult patients in intensive care units [24] and in children with febrile neutropenia [25]. A recent study evaluated 49 children, who had 60 febrile episodes compared follow-up value of PCT with CRP in documenting the infection in neutropenic febrile patients. PCT and CRP levels were significantly higher in neutropenic febrile patients with infection than in control patients (P < 0.001). In sequential analyses of patients without documented infections, the median of PCT concentrations shows a tendency to fall after the 8th hour of onset of fever, whereas in patients with documented infections PCT concentrations fell after the 48th hour. This study suggests that PCT, when measured periodically, is a more useful diagnostic inflammation parameter in pediatric neutropenic-fever patients than CRP [26].

In our study, when we evaluated prognosis, its association with platelet count, even though significant from a statistical point of view, is not useful taking into account that it does not present a large increase of the probability of death as an outcome.

Although Giamarellou *et al *(2004) found a area under the ROC curve of 71%, very close to the one of these study, the present data suggest a lower cut-off value for PCT (0.5 ng/mL) could distinguish infected neutopenic febrile patients from those without severe systemic infection.

## Conclusion

PCT concentration was markedly associated with the diagnosis of severe infection in patients with febrile neutropenia, in contrast with CRP whose concentration was so useful to distinguish between presence and absence of a disseminated infection. None of the other variables of the study could be correlated to prognosis.

Further studies, with larger samples, involving PCT concentration curves and with and more homogeneous groups of febrile severely neutropenic patients should be carried out in other centers in order to confirm our results.

## Competing interests

The author(s) declare that they have no competing interests.

## Authors' contributions

KSRM conceived and design the study and carried out the assay of the PCT, SFC participated in the design and interpretation of the study, CL performed the statistical analysis, DAFC participated in its coordination. All authors read and approved the final manuscript.

## Pre-publication history

The pre-publication history for this paper can be accessed here:


